# Influence of Accumulative Roll Bonding on the Texture and Tensile Properties of an AZ31 Magnesium Alloy Sheets

**DOI:** 10.3390/ma11010073

**Published:** 2018-01-05

**Authors:** Zuzanka Trojanová, Ján Džugan, Kristýna Halmešová, Gergely Németh, Peter Minárik, Pavel Lukáč, Jan Bohlen

**Affiliations:** 1 Department of Physics of Materials, Faculty of Mathematics and Physics, Charles University, Ke Karlovu 5, 121 16 Praha 2, Czech Republic; ztrojan@met.mff.cuni.cz (Z.T.); gergely1227@gmail.com (G.N.); peter.minarik@mff.cuni.cz (P.M.); lukac@met.mff.cuni.cz (P.L.); 2 COMTES FHT a.s., Průmyslová 995, 334 41 Dobřany, Czech Republic; jdzugan@comtesfht.cz; 3 Helmholz Zentrum Geesthacht, Magnesium Innovation Centre, Max Planck Street 1, D21502 Geesthacht, Germany; jan.bohlen@hzg.de

**Keywords:** magnesium alloy, accumulative roll bonding, texture, tensile anisotropy, twinning, dynamic recrystallization

## Abstract

Deformation behaviour of rolled AZ31 sheets that were subjected to the accumulative roll bonding was investigated. Substantially refined microstructure of samples was achieved after the first and second pass through the rolling mill. Sheets texture was investigated using an X-ray diffractometer. Samples for tensile tests were cut either parallel or perpendicular to the rolling direction. Tensile tests were performed at temperatures ranging from room temperature up to 300 °C. Tensile plastic anisotropy, different from the anisotropy observed in AZ31 sheets by other authors, was observed. This anisotropy decreases with an increasing number of rolling passes and increasing deformation temperature. Grain refinement and texture are the crucial factors influencing the deformation behaviour.

## 1. Introduction

Magnesium alloys are promising lightweight materials due to their high specific strength and specific stiffness (ratio of the strength/stiffness to density). A significant factor is also the fact that there is an abundance of resources of these alloys. Magnesium alloys of the AZ (Mg-Al-Zn) series exhibit further benefits, such as excellent castability, good corrosion resistance, and good recyclability [1]. The strength of AZ magnesium alloys for increases with increasing Al content while ductility decreases. This is reason why the AZ91 alloy is used in as-cast state, while the AZ31 alloy is considered as a wrought material. AZ31 alloys sheets are manufactured usually by rolling at elevated temperatures [2,3]. Deformation of polycrystals with lower symmetry up to the large strains is a major issue, which has not been satisfactorily solved up to now. It is well known that hexagonal close packed (hcp) magnesium alloys exhibit limited formability in comparison with cubic face or body centered metals. The main deformation systems of pure magnesium and hcp magnesium alloys are composed of three glide systems (basal, prismatic, and pyramidal slip), which are not crystallographic equivalent and mechanical twinning. At room temperature, the critical resolved shear stress (CRSS) of the basal system is much lower than the CRSS of non-basal and twinning systems [4]. As a result, the plasticity of almost all cast magnesium alloys is limited because von Mises criterion is not met [5]. This situation changes at temperatures above 200 °C where the CRSS of non-basal slip systems became comparable with the CRSS of the basal system [6]. In the accumulative roll bonding (ARB) process, as firstly described in [7], two sheets of a material are purified and joined together by rolling. Then, the rolled material is cut into two halves again surface treated and roll-bonded again. This process may be repeated several times. In the case of magnesium alloys, the number of passes through the rolling mill is limited to 1–3 passes due to the limited plasticity of the hcp structure [8]. Originally, ARB process should be performed at temperatures lower than the recrystallization temperature [9]. It is possible in fcc and bcc metals, such as aluminum, copper, and steel [10,11,12]. In hcp metals, such as zirconium, titanium, and magnesium higher temperatures are required for good formability [13,14,15]. Microstructure and texture evolution of Mg-Al-Zn alloys submitted to ARB process were reported in several papers [16,17,18,19]. Mechanical properties of conventionally rolled magnesium alloys sheets usually exhibit a significant planar plastic anisotropy at room temperature [20,21]. The tensile yield strength measured in the rolling direction is usually lower in comparison to the strength measured in the transversal direction, as a result of the texture formation during the processing. Planar anisotropy of the magnesium alloy sheets submitted to the ARB process exhibit also other physical properties such as elastic modulus and thermal expansion coefficient [22].

In this paper, we investigate AZ31 sheets after ARB with the aim to reveal microstructure and texture evolution after one and two ARB passes, as well as the effect of microstructure and texture on the mechanical behaviour, including mechanical anisotropy at room and elevated temperatures.

## 2. Material and Methods

**Figure 1:**
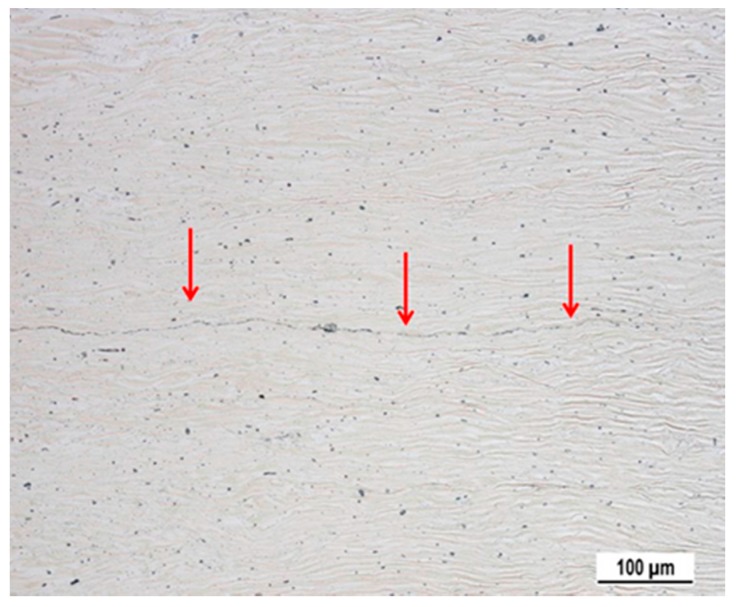
New interface formed in the sample after the first rolling pass. Micrograph performed in SEM Tescan.

Commercially available sheets of AZ31 alloy were used in this study. Sheets were supplied by the Kalbin Metals Co. (Tijanjin, China). The chemical composition of the alloy is given in Table 1. Four-high configuration of rolling mill (PZSK, Kladno, Czech Republic) (i.e., two backing and two working rolls) was used for production of ARBed sheets. Two as-received sheets with an initial thickness of 2 mm were wire brushed then cleaned with acetone and riveted along one side to ensure sufficient roughness and absence of oxides to make quality joint. The ARB process was carried out with a rolling speed of 0.4 mm/s and 50% reduction in the thickness per cycle with no lubrication applied. The rolling process was carried out at 350 °C and 400 °C. At 350 °C materials failed to bond and at 400 °C we got good quality joins up to 2 passes through the rolling mill. Before rolling, the sheets were heated up to 400 °C that is typical temperature for hot rolling practice. We performed one and two cycles of the ARB process. Excessively high total reduction resulted in edge cracks. Longitudinal cross section of samples, normal to the transverse direction, was observed in the light microscope. The joining of both sheets is partially visible, as it is shown in the Figure 1.

Oxide and precipitate particles are dispersed uniformly along the interface. After the second rolling pass, the material exhibits three new interfaces. The bonding at the interface was very good, debonding or delamination at the interface was not observed even during deformation experiments at elevated temperatures. 

The equivalent von Mises strain which was achieved during accumulative roll bonding process can be expressed [23]

(1) $$\epsilon =\;\left[\frac{2}{\sqrt{3}}\;ln\left(\frac{1}{5}\right)\right]\;n$$

where *n* is number of passes. It means that the equivalent strain ϵ=1.86 after the first pass and ϵ = 3.72 after the second pass was introduced to the material.

Microstructure of samples was studied using scanning electron microscopes (SEM) Tescan (TESCAN ORSAY HOLDING, Brno, Czech Republic) and ZEISS Auriga Compact (Carl Zeiss Microscopy, Jena, Germany) equipped with EDAX EBSD camera; OIM software was utilized for EBSD observations. Samples were first mechanically grinded then polished with a diamond suspension of the grade 3, 1, and ¼ µm and alumina suspension of the grade 0.05 µm. Finally, the samples surface was ion-polished by Leica EM RES102 (Leica Mikrosysteme, Wetzlar, Germany) device in order to get a high quality surface for EBSD measurements. The texture was determined using an X-ray PANalytical XPert MRD diffractometer (PANanalytical, Almelo, The Netherlands) using CuK_α_ radiation from the sheets surface. The investigated diffractions were (0002), $\left(10\bar{1}0\right)$,$\;\left(10\bar{1}1\right)$,$\;\left(10\bar{1}2\right)$,$\;\left(10\bar{1}3\right)$,$\;\left(11\bar{2}0\right)$, from which the full pole figures were calculated using MTEX software [24]. Acoustic emission (AE) activity was followed using DAKEL-IPL (DAKEL, Rpety, Czech Republic) system with the four channel detection and continuous storage of AE signals with a 2 MHz sampling frequency. The AE signal threshold level of 26 dB was used to detect the burst AE activity, observed mainly as a consequence of an instable plastic deformation. 

**Figure 2:**
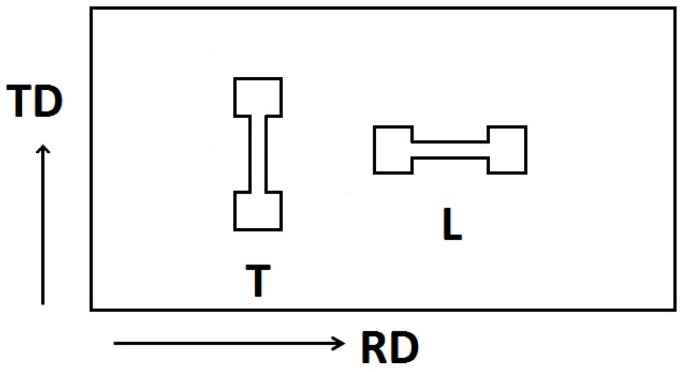
Geometry of samples for tensile tests.

Samples for tensile tests had a dog-bone shape with a gauge length of 20 mm. Samples were cut from the sheets so that the deformation axis was parallel to the rolling direction (L) and perpendicular to the rolling direction (T), see Figure 2. Samples of both orientations were tested using screw-driven deformation device Instron 5882 (INSTRON, High Wycombe, UK) at temperatures ranging from room temperature up to 300 °C, with an initial strain rate of 1 × 10^−3^ s^−1^. The true stress—true plastic strain curves were calculated. Characteristic stresses, i.e., the tensile yield strength (TYS) and ultimate tensile strength (UTS) were estimated together with the plastic strain to fracture, ε_f_. TYS was estimated as the offset stress for the plastic strain of ε = 0.002. Samples cut from as received sheet and after one or two passes through the rolling mill are marked hereafter ARB_0, ARB_1, and ARB_2.

## 3. Results

### 3.1. Microstructural Characterisation

**Figure 3:**
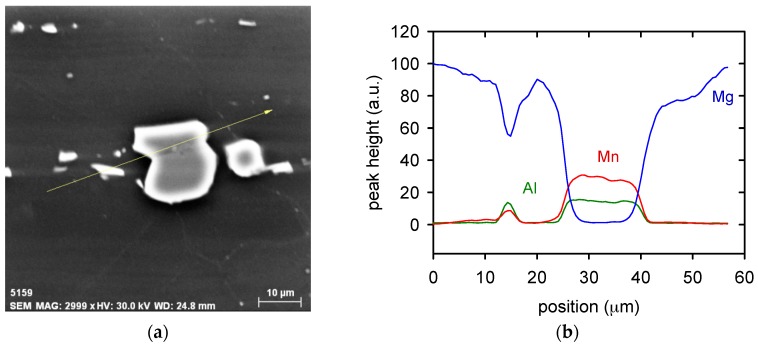
Particle in the microstructure (**a**) and its chemical composition (**b**).

**Figure 4:**
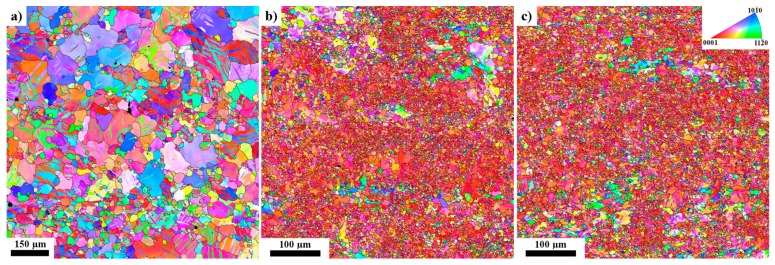
EBSD microstructure of the investigated samples. (**a**) ARB_0; (**b**) ARB_1 and (**c**) ARB_2.

SEM analysis revealed that the small black particles observed in a high density in Figure 1 contain Al and Mn, as it is documented in Figure 3. The particles are the most probably Al_8_Mn_5_ precipitates, which are typically observed in AZ31 alloy [25]. The effect of ARB on the sheet grain structure is shown in Figure 4 in the means of EBSD. The pictures were taken from the sheet surface. The micrograph of the as-received material is shown in Figure 4a. Large grains of size ~100 µm dominate the microstructure. Additionally, a high number of twins are present in the microstructure. The first ARB pass resulted in a significant change of the materials microstructure, see Figure 4b. The average grain size decreased by one order to the magnitude of ~10 µm. The highest area fraction had grains of 4–5 µm in diameter; nevertheless, still there were areas of much bigger grains (40–60 µm). The fraction of high angle grain boundaries was particularly high ~0.8. However, the variation of the colour is visible mostly in the large grains because of a high lattice distortion density. This is evidence that grains interiors are severely plastically deformed with the high stored energy. 

**Figure 5:**
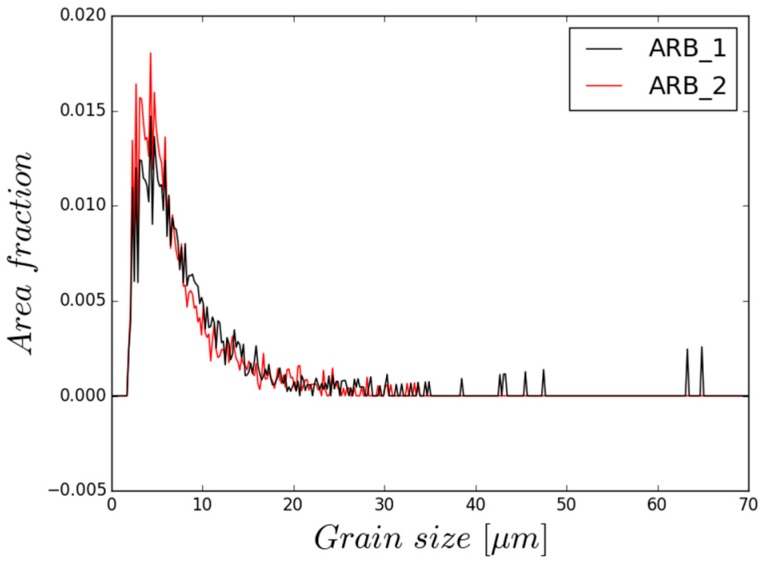
Grain size distribution of ARB_1 and ARB_2 sample calculated from EBSD data.

The microstructure of sample processed by two passes of ARB is shown in Figure 4c. Additional processing by ARB resulted more in homogenisation of the microstructure rather than in further grain refinement, as shown in Figure 5. As a result, the average grain size decreased to ~7.6 µm, but the highest area fraction was still represented by grains of 4–5 µm in diameter. It is important to note that the large grains (>30 µm) practically vanished from the microstructure. 

The grain refinement during high temperature rolling took place via the operation of the rotational dynamic recrystallization (RDX). This mechanism involves the dynamic polygonisation of rotated lattice regions that are adjacent to the grain boundaries [17,26]. This assumption is particularly supported by high deformation energy stored in larger grains. RDX very probably may also contribute to the joining of sheets during the ARB process [27]. 

**Figure 6:**
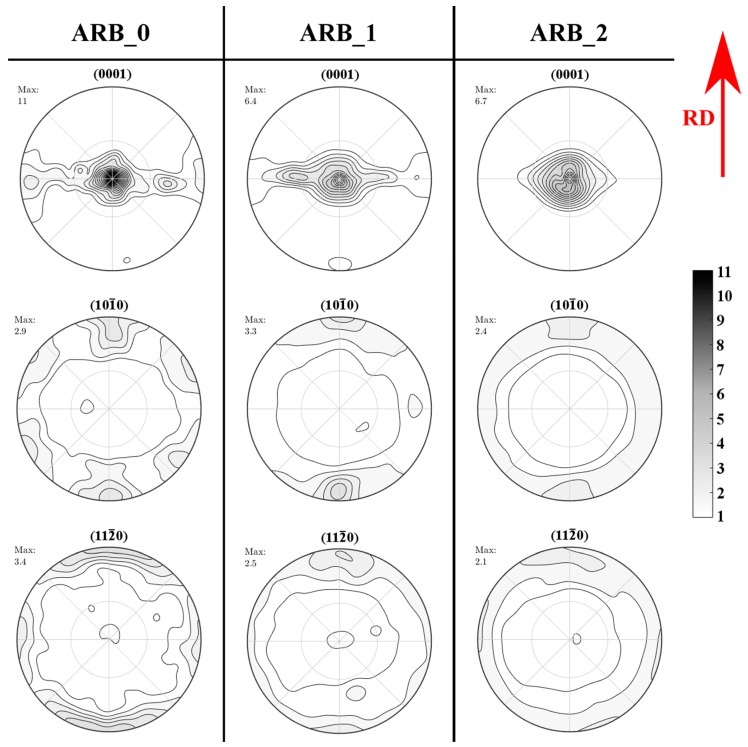
XRD pole figures of the investigated samples.

The XRD pole figures, as shown in Figure 6, document the texture evolution as a result of the ARB processing. The as-received material was in a form of rolled sheet with a typical (0001) basal texture. Nevertheless, severe splitting up to 90° towards the transverse direction was observed in the (0001) pole figure. Processing by ARB resulted in a significant weakening of the observed splitting after the first pass. The central maximum became less sharp and a decrease of the overall texture strength was measured. The second pass of ARB caused a further sharpening of the basal texture. Splitting along the transverse direction was only limited in this sample, but the overall texture strength remained almost the same. The texture observed in the as-received material differs from the typical texture of the AZ31 sheets that are presented by other authors [28,29].

The as-rolled basal texture in magnesium is usually sharp with majority of c-axes aligned along the sheet normal direction and splitting of basal pole is usually observed along the rolling direction. As a result, observed texture development during ARB processing is strongly affected by the initial texture and the processing cause reorienting of grains closer to the typical texture of the rolled AZ31 alloy. 

### 3.2. Tensile Tests

**Figure 7:**
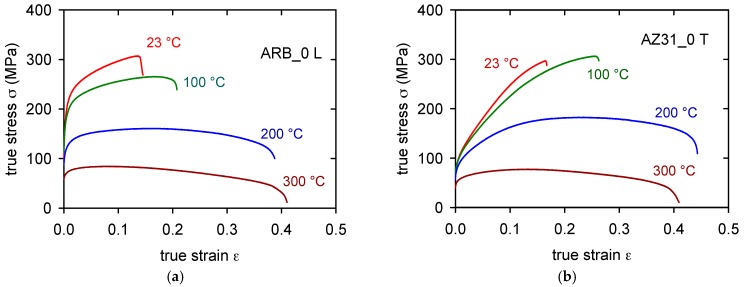
True stress-true strain curves obtained for four temperatures of ARB_0 samples for L-orientation (**a**) and T-orientation (**b**).

The true stress-true strain curves obtained for the as-received sheet samples (ARB_0) cut in the longitudinal direction (L) and transversal direction (T) at various temperatures are plotted in Figure 7a,b. Differences between both sets of curves are obvious: the flow stresses for samples of the L-type are higher than those for the T-samples. On the other hand, the strain to fracture, ε_f_, is a bit higher for curves that are obtained at temperatures higher than 100 °C (see Table 2). The strain to fracture is comparable for both sampling orientations at temperatures above 100 °C. Characteristic stresses (TYS and UTS) are summarized for various temperatures in Figure 11a–d. The significant anisotropy of the TYS is obvious for all the temperatures. 

The TYS for the L-orientation is higher than TYS for the T-orientation at all temperatures, even when the difference decreases with increasing temperature. Curve shapes of L-samples and T-samples obtained at 23 and 100 °C are different. While L-curves are flat, the T-curves exhibit much higher strain hardening rate.

**Figure 8:**
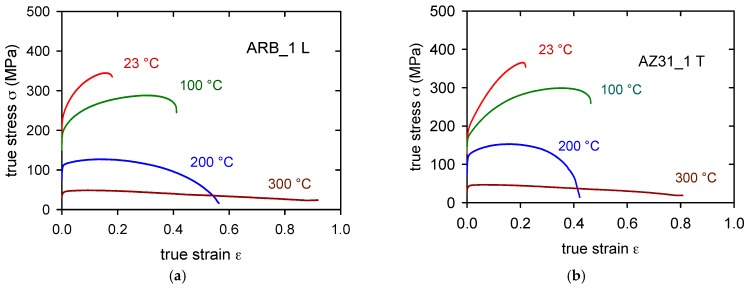
True stress—true strain curves obtained at various temperatures for L-samples (**a**) and T-samples (**b**) after one pass through the rolling mill.

The true stress-true strain curves obtained at various temperatures for ARB_1 samples for both sampling orientations are presented in Figure 8a,b. It is clear that deformation stresses are much higher when compared with the ARB_0 sample. Plasticity of both sample types, L and T, increased comparing with the ARB_0 samples at all temperatures studied. As it can be seen in Figure 11, the anisotropy of samples decreases with increasing temperature; at temperatures 200 and 300 °C, the TYS of L- and T-samples are comparable.

**Figure 9:**
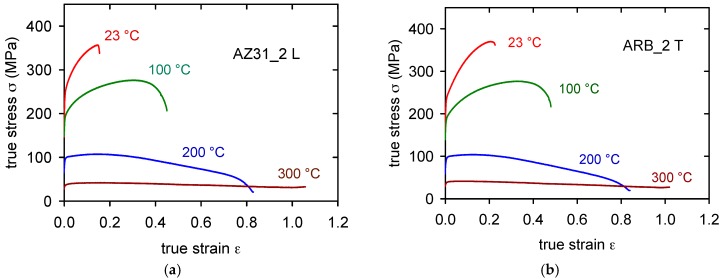
True stress—true strain curves obtained at various temperatures for L-samples (**a**) and T-samples (**b**) after two passes through the rolling mill.

The true stress-true strain curves obtained for ARB_2 samples are shown in Figure 9a for L-samples and Figure 9b for T-samples. Anisotropy of samples again decreased for lower temperatures, at temperatures higher TYSs for both of the samples are comparable. 

**Figure 10:**
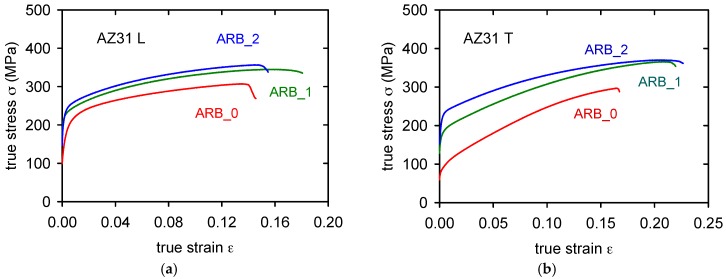
True stress—true strain curves obtained at room temperature for various numbers of passes: (**a**) L-samples, (**b**) T-samples.

Plasticity at 300 °C is higher than 100% (see Table 2). The observed increase of the flow stresses at room temperature after the first and second rolling passes is visible in Figure 10a for L-type samples and in Figure 10b for T-samples. An observed increase of the true stresses between the first and second passes, for both types of samples, indicate that the effect of the accumulative roll bonding has very probably its saturation level. The stress increase is higher in the case of T-samples. Ductility of samples is relatively high, about 15% in the case of L-samples and 17–23% in T-samples. Anyway, the plasticity of both L- and T-samples is relatively high at room temperature when comparing with as-cast magnesium materials that usually achieve only units of percent. Values of the fracture strain, ε_f_, are for both types of samples and all test temperatures reported in Table 2.

## 4. Discussion 

The mechanical properties of AZ31 alloy sheets after accumulative roll bonding are influenced with three principal factors: the grain size, texture, and dislocation density. Figure 4a–c show that the ARB process resulted into significant grain refinement. Pawar et al. [8] studied role of Al_8_Mn_5_ particles on microstructure of a twin roll cast AZ31 alloy. They found that these very fine particles act as the nucleation centres in the α -Mg grains in the recrystallisation process. Material strengthening due to grain size refinement may be expressed by the Hall-Petch relationship:

(2) $$\sigma_{\varepsilon }=\sigma_{0\varepsilon \;}+\;k_{\varepsilon }\;d^{-1/2},$$

where σ_ε_ is the true stress at true strain ε, σ_0ε_ is friction stress, *k*_ε_ is microstructural stress intensity, and *d* is average grain diameter. The value of ε may be the initial yield strain or proof strain that is determined for yielding or the subsequent strain for a plastic flow stress that is finally terminated in fracturing [30]. In the dislocation pile-up model, *k*_ε_ is related to the concentration stress, τ_C_, as

*k*_ε_ = *m*_T_ (π*m*_S_ G*b*τ_C_/2 α)^1/2^ (3)

In Equation (3), *m*_T_ and *m*_S_ are Taylor and Sachs orientation factors, G shear modulus, *b* magnitude of the Burgers vector, and α, a numerical factor following from the mutual dislocation interactions. In the first approximation, it is possible to take for α ≈ 1. The *k*_ε_ term was experimentally estimated for the AZ31 alloys. Data reported by various authors differs in a wide range [31,32,33]. A reasonable value for *k_y_* ≈ 10 MPa·mm^1/2^ indicates a relatively strong sensitivity of the AZ31 alloy to the grain size strengthening and the fact that the mechanism of pile-ups formation plays an important role in the deformation process.

**Figure 11:**
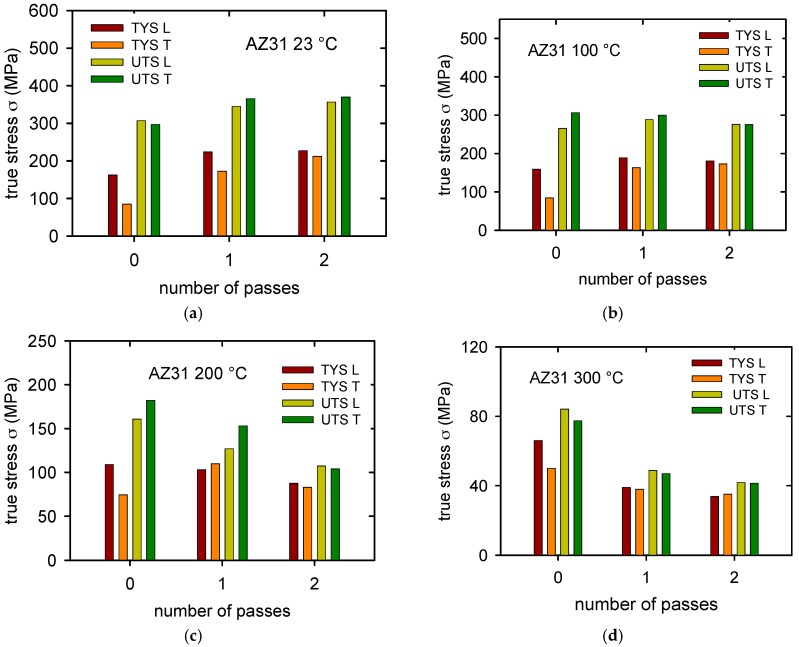
Characteristic stresses obtained for samples in L- and T-orientation after 0, 1 and 2 rolling passes at (**a**) room temperature; (**b**) 100 °C; (**c**) 200 °C and (**d**) 300 °C.

Observed plastic anisotropy is well visible in Figure 11. Stress that is necessary for plastic deformation of the T-samples is lower when compared with the flow stresses that were found for the L-samples. This anisotropy was found for all deformation temperatures as it follows from diagrams shown in Figure 11. The ARB process weakened this anisotropy. In the samples after two rolling passes the difference between the TYSs is smaller and at the highest temperature of 300 °C the yield strengths of samples with different orientations are comparable. Character of the observed plastic anisotropy differs from results reported in published papers. Authors of [20,21] found that the TYS is lower for the samples oriented in the rolling direction compared with samples oriented in transversal direction. Our results have shown that the TYS obtained in the rolling direction is higher when compared with the TYS in the transversal direction. This fact very probably follows from the texture that was found in the as-received samples and after the ARB process. The texture can be characterised as a basal texture with a majority of **c**-axes perpendicular to the sheet surface; moreover, severe splitting of basal poles into transversal direction was observed (see Figure 6). Such type of the texture with the angular distribution of the basal poles broader in the transversal direction was observed in Mg alloys containing rare earths or Ca [34]. In the literature, it has been reported that even small (0.035%) Ce addition changes the texture of Mg alloys so that the splitting of the basal poles is in the transversal direction [34,35,36]. TYS of such alloys is lower in the transversal direction when compared with the TYS estimated in the rolling direction. The “unusual” texture observed in the studied AZ31 alloy is very probably a consequence of the small addition of Ce, as it is documented in the chemical composition reported in Table 1. It is known that $\left\{10\bar{1}2\right\}$ tensile twins are formed in magnesium and magnesium alloys, in a tensile experiment, when the <c> axis of the hexagonal cell is parallel to the stress axis [37,38,39].

**Figure 12:**
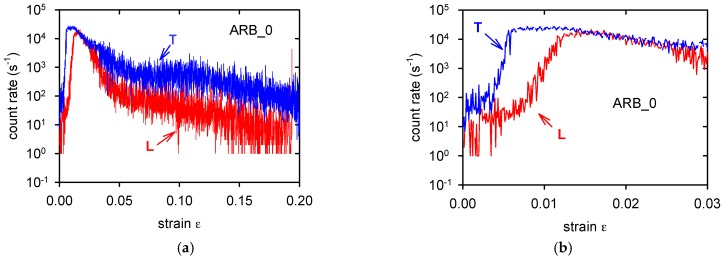
Acoustic emission rate measured during deformation of the ARB_0 samples (**a**). Beginning of the plastic deformation is recorded in (**b**).

Observed splitting of the (0001) poles into the transversal direction indicates that there are grains in the T-samples exhibiting nearly parallel orientation of the <c> axis to the tensile axis. We may consider that different deformation mechanisms that are operating in samples with the L- and T-orientations are responsible for different characteristic stresses, as estimated in tensile experiments. While deformation of L-samples is mainly realised by the dislocation glide, mechanical twinning plays an important role during deformation of T-samples, especially at the beginning of the deformation process. This statement was supported by the acoustic emission (AE) record during deformation of ARB_0 samples, as shown in Figure 12.

**Figure 13:**
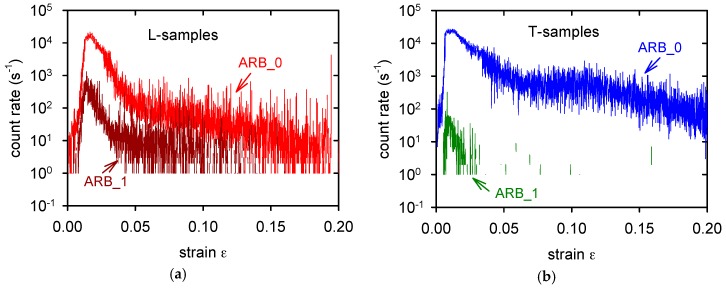
Acoustic emission rate measured during deformation of the L-samples (**a**) and T-samples (**b**).

AE from deformed T-samples was more intensive, as it is documented in Figure 12a. The difference between AE of the L- and T-samples at the onset of deformation process is better visible in Figure 12b. Mainly at the beginning of the deformation process, the AE signal is emitted at the earlier stages of deformation and it has intensity that is 10 times higher. It is known that the twins’ formation is an effective source of AE signal [40]. Activity of twinning decreases in Mg alloys with an increasing deformation temperature; for temperatures that are higher than 473 K is not significant [40]. In the first and second rolling pass, the basal texture was distinguished and the splitting of basal poles towards transverse direction was not so pronounced. It is very probably one of reasons why the plastic tensile asymmetry of ARB_1 and ARB_2 samples was not so strong. The second reason why the samples anisotropy decreases with the number of passes lies in the decreasing grain size. Analogous relationship to the Hall-Petch equation may also be written for twinning [41,42]

(4) $$\sigma_{t}=\;\sigma_{0t}+k_{t}d^{-1/2}$$

where indices *t* are related to twinning. Somekawa and Mukai [42] have shown that the Hall-Petch constant *k_t_* for twinning is higher when compared with the *k_d_* for slip deformation. The influence of the grain size on the twinning stress is much stronger [43]. It was also confirmed with the AE measurements. AE signal generated from deformed ARB_1 samples was much weaker than the one detected for the ARB_0 samples, as it is documented in Figure 13a for L-samples and Figure 13b for T-samples. No AE signal was detected during the plastic deformation of ARB_2 samples.

Mechanical work consumed during plastic deformation is converted into the energy of newly created crystal defects and heat. Such stored defects may be dislocations, twins, grain boundaries, point defects, and other interfaces in the material. When considering isothermal deformation, performed at low strain rates, possible temperature increase in the sample may be neglected. Although the grain refinement is the important strengthening term, a high dislocation density present in the severely deformed materials may contribute significantly to the strengthening of ARB sheets. The stress, σ, necessary for deformation is related to the total dislocation density, ρ*_t_*, by the known Taylor relation [44].

(5) $$\sigma =m_{T}\alpha_{1}Gb\rho_{t}^{1/2}$$

where *m_T_* is the Taylor factor, *G* shear modulus and *b* the Burgers vector of dislocations. Factor α_1_ was estimated for hexagonal metals by Lavrentev α_1_ = 0.35 [45]. Starink analysed correlation between grain size and dislocations strengthening in severaly deformed metallic materials [46]. He found that the dislocation strengthening is more important that the grain boundary strengthening. From Figure 7, Figure 8 and Figure 9, it can be seen that the strain hardening takes place at temperatures up to 200 °C.

Strain hardening of a material is influenced mainly by an increase of the dislocation density, storage of dislocations inside grains and dislocation interactions. On the other hand, softening processes are present especially at elevated temperatures. Then, the work hardening rate is a result of two principal competitive processes—hardening and softening. Trojanová and Lukáč analysed these processes operating in Mg alloys [47]. Interaction between basal <a> dislocations and non-basal <a + c> dislocations may form sessile <c> dislocations with the Burgers vector perpendicular to the slip plane. Such dislocations are not able to move conservative by the slip, they can only climb. This is possible only at elevated temperatures. Grain boundaries and twins are further obstacles for dislocation motion which contribute to the strain hardening. With the decreasing grain size the density of grain boundaries increases too. Twins formation is massive at the beginning of the deformation process, as it was mentioned above. Newly formed twins are effective obstacles for dislocation motion and contribute to the strain hardening. This is clearly demonstrated on the stress-strain curve that is obtained at room temperature for the T-sample. Rapid stress increase indicates that the work hardening rate is higher when comparing with the flat curve measured for the L-sample. Cross slip and dislocation climb may be considered as the main softening processes. An increase in the activity of non-basal slip systems with increasing temperature contributes to the enhanced ductility at elevated temperature. Máthis et al. [6] studied the relation of non-basal to basal dislocations in the deformed Mg. They estimated an increased fraction of non-basal dislocations at higher temperatures on the account of basal dislocations. Mobility of non-basal dislocations may be influenced by the alloying elements. Mishra et al. [36] estimated that the Ce addition facilitates the motion of non-basal dislocation even in very small concentrations which does not influence the <c>/<a> ratio. Higher characteristic stresses were observed at temperatures 23 and 100 °C for ARB_1 and ARB_2 samples when compared with ARB_0 samples. At temperatures higher than 100 °C mechanical properties of ARB_0 samples are the best. It is due to the fact that the grain boundary sliding mechanism is active at elevated temperatures in fine grained materials (ARB_1 and ARB_2). This mechanism takes place at elevated temperatures when the grain size is lower than 10 µm [48]. This deformation mode is also supported by the thermally activated diffusion mechanism which is more intensive at elevated temperatures. 

## 5. Conclusions

AZ31 magnesium alloy sheets were processed by the accumulative roll bonding. Microstructure and texture of samples submitted one and two rolling passes were studied. Tensile properties of materials were investigated at room and elevated temperatures. Following conclusions result from this complex study:

- During the ARB procedure, the sheets grain size was drastically decreased.
- ARB sheets exhibit (0001) basal texture with the split of basal poles in the transversal direction.
- A significant improvement of mechanical properties at room temperature was observed after the first and second rolling passes.
- Tensile yield strength measured in the rolling direction is higher than that estimated in the transversal direction.
- This plastic anisotropy decreases with increasing number of passes and with increasing deformation temperature.
- Different deformation mechanisms are responsible for the anisotropy observed particularly at temperatures lower than 200 °C. While the straining of samples oriented in the rolling direction takes place mainly by the dislocation mechanism, twinning operates at the beginning of the plastic straining in the transversal direction.
- Plastic deformation at 200 and 300 °C is influenced in the fine grained ARB_1 and ARB_2 samples with the grain boundary sliding.

## Figures and Tables

**Table 1:** Chemical composition of the sheet (in wt.%).

Al	Zn	Mn	Si	Fe	Ce	Mg
3.2	1.3	0.4	0.01_5_	0.03	0.06	Bal.

**Table 2:** Strain to fracture, ε_f_, estimated for both samples orientations and temperatures.

Material	L-orientation				T-orientation			
T/°C	23	100	200	300	23	100	200	300
ARB_0	0.146	0.208	0.388	0.410	0.167	0.263	0.443	0.410
ARB_1	0.181	0.412	0.563	0.918	0.220	0.464	0.422	0.808
ARB_2	0.155	0.450	0.829	1.057	0.227	0.480	0.840	1.020
